# Comparison of spectral-domain optical coherence tomography biometry measurements with partial coherence interferometry optical biometry

**DOI:** 10.1186/s12886-026-04979-3

**Published:** 2026-06-03

**Authors:** Konuralp Yakar, Gökhan Özgür

**Affiliations:** 1 Department of Ophthalmology, Faculty of Medicine, Atılım University, Ankara, Turkey; 2 Department of Ophthalmology, Medicana International Samsun Hospital, Samsun, Turkey

**Keywords:** AL Scan, Biometry, REVO FC, OCT

## Abstract

**Purpose:**

This study aimed to evaluate the agreement and interchangeability between a spectral-domain optical coherence tomography (SD-OCT)-based biometer (REVO FC) and a partial coherence interferometry (PCI) device (AL Scan).

**Methods:**

This prospective comparative cross-sectional study included 60 right eyes of 60 patients aged 40–82 years, diagnosed with senile cataracts. Measurements of axial length (AL), anterior chamber depth (ACD), keratometry (K1, K2), and astigmatism vector components (J0, J45) were conducted using both devices. Paired comparisons were performed using appropriate statistical tests. Agreement was evaluated using Bland–Altman analysis, while correlation and reliability were assessed using Pearson correlation coefficients and interclass correlation coefficients (ICC).

**Results:**

No statistically significant differences were observed in AL (*p* = 0.507) or ACD (*p* = 0.218), with excellent correlations (AL: *r* = 0.998; ACD: *r* = 0.945). Bland–Altman analysis demonstrated excellent agreement for AL measurements, with a mean difference (bias) of − 0.01 mm and narrow 95% limits of agreement (LoA: −0.245 to 0.225 mm). For ACD, the mean difference was 0.02 mm, with a moderately wider LoA (− 0.244 to 0.288 mm), indicating an acceptable but less robust agreement compared to AL. The keratometric values were significantly higher with REVO FC (K1: +0.76 D; K2: +0.94 D; both *p* < 0.001), indicating a systematic bias. Despite strong correlations (K1: *r* = 0.980; K2: *r* = 0.924), the Bland–Altman analysis revealed wide limits of agreement. J0 exhibited no significant difference (*p* = 0.889), whereas J45 differed significantly (*p* = 0.028). Both vector components demonstrated moderate agreement, with relatively wide limits of agreement.

**Conclusion:**

REVO FC and AL Scan demonstrate excellent agreement for AL and ACD measurements and may be used interchangeably for these parameters. However, significant discrepancies in keratometric and astigmatic measurements limit their interchangeability.

## Introduction

Accurate ocular biometry is essential for the precise calculation of intraocular lens (IOL) power and optimal refractive outcomes in contemporary cataract and refractive surgeries. Optical biometry has largely supplanted ultrasound-based techniques because of its superior precision, repeatability, and non-contact measurement capability [1, 2]. Utilizing partial coherence interferometry (PCI), swept-source, or spectral-domain optical coherence tomography (SD-OCT), these devices facilitate the high-resolution measurement of critical parameters such as axial length (AL), anterior chamber depth (ACD), keratometry (K1, K2), and central corneal thickness (CCT). Enhancements in biometry accuracy have significantly diminished postoperative refractive surprises and improved surgical predictability [3, 4].

The AL Scan is a PCI-based optical biometer that integrates Scheimpflug imaging and optical interferometry to rapidly acquire multiple ocular parameters with high repeatability. Previous studies have demonstrated excellent intra-device reproducibility (intraclass correlation coefficients [ICC] often > 0.90) and strong concordance with established biometers such as Lenstar and IOLMaster [5, 6]. Additionally, AL Scan measurements of keratometry, axial length, and corneal thickness have shown a high correlation with other modalities, supporting its clinical reliability [7].

More recently, SD-OCT–based biometers, such as the REVO FC (Optopol Technology), have emerged, combining anterior segment OCT imaging with biometry. These systems offer micrometer-resolution cross-sectional imaging and enable the direct visualization of ocular structures alongside biometric acquisition. Studies evaluating SD-OCT biometers have reported excellent repeatability (ICC up to 0.999 for axial length) and high measurement consistency, suggesting their potential as next-generation devices for ocular biometry [8, 9].

Despite the widespread use of optical biometers, the interchangeability between devices remains a critical issue. Even minor systematic differences in biometric measurements may lead to clinically significant refractive errors, particularly in premium IOL implantation. While several studies have compared PCI-based biometers with other technologies, data comparing SD-OCT–based systems with established devices such as the AL Scan remain limited [10, 11]. Furthermore, differences in measurement principles, such as interferometry versus OCT-based imaging, may introduce variability in biometric outputs.

This study aimed to assess the agreement and interchangeability of the measurement results for AL, ACD, keratometry readings (K1, K2), and astigmatism vector analysis (J0 and J45) obtained using the REVO FC and AL Scan devices.

## Methods

This prospective, comparative, cross-sectional study included the biometric data of 60 right eyes of 60 patients aged 61.98 ± 10.69 years (range, 40–82 years) who visited our ophthalmology clinic and were diagnosed with senile cataracts between January 2026 and March 2026. This study was approved by the Institutional Review Board of XXXX (Approval No: GOKAEK 2025/11/10) and adhered to the principles outlined in the Declaration of Helsinki. Written informed consent was obtained from all participants after a detailed explanation of the study and its procedures. Each patient underwent a thorough eye examination by the same physician, which included tests for visual acuity, measurements of intraocular pressure, and evaluations of both the anterior and posterior segments. Individuals who had previously undergone ocular surgeries, including refractive surgery, pterygium excision, glaucoma surgery, keratoplasty, or vitrectomy, were excluded from the study. Additionally, those with acquired or hereditary corneal pathologies, active keratitis or conjunctivitis, corneal scarring, nystagmus, albinism, or any corneal, vitreous, or retinal conditions that could potentially influence the biometric measurements were also excluded. AL, ACD, flat and steep keratometric measurements (K1, K2), and J0–J45 vector values (Jackson crossed cylinder components with powers at axes 180° and 45°) were compared. These measurements were performed prior to pupil dilation according to the device manufacturer’s guidelines. All measurements were performed by the same, experienced technician. The J0 and J45 parameters were calculated as follows:

*C (Cylinder)=K2-K1,\,Axis=K1Axis*

*J0=(-C / 2)xCos(2xAxis)*

*J45=(-C/ 2)xSin(2xAxis)*

## Instruments

The REVO FC (Optopol Technology, Zawiercie, Poland) is a SD-OCT device that combines anterior segment imaging and optical biometry within a unified platform. The system utilizes a superluminescent diode light source with a wavelength of approximately 870 nm and achieves a scanning speed of up to 80,000 A-scans/s. Through OCT-based boundary detection, the device identifies ocular interfaces along the visual axis, facilitating the measurement of various biometric parameters, including AL, ACD, CCT, LT, pupil diameter, and white-to-white corneal diameter (WTW). Keratometry measurements were derived from the OCT-based anterior corneal surface data. The curvature of the anterior corneal surface was calculated from the segmented OCT images, and keratometric values (K1, K2, and astigmatism axis) were obtained from the calculated corneal radius using a standard keratometric refractive index on a 3 mm zone. The system enables the visualization of ocular structures in cross-sectional OCT images and provides axial measurements with micrometer-level precision.

The AL Scan (NIDEK Co., Gamagori, Japan) is an optical biometer that uses PCI for axial measurements in the ocular biometry. This device facilitates the rapid acquisition of multiple biometric parameters, including AL, corneal curvature (keratometry), ACD, CCT, white-to-white WTW, and pupil size, typically within approximately 10 s of use. Keratometry was conducted using a double-mire ring reflection keratometry system, wherein concentric rings of light were projected onto the anterior corneal surface, and their reflected images were analyzed to calculate the radius of the corneal curvature. The system assesses corneal curvature using measurements from two concentric zones, approximately 2.4 mm and 3.3 mm in diameter, enabling the calculation of flat keratometry (K1), steep keratometry (K2), and the astigmatism axis. The device incorporates 3-D auto-tracking and auto-shot alignment, which monitor eye movements along the X-, Y-, and Z-axes to ensure precise alignment and automated image capture during measurement. Additionally, the system includes Scheimpflug imaging of the anterior segment, which aids in the evaluation of anterior ocular structures and supports measurements, such as the anterior chamber depth.

## Statistical analysis

Statistical analyses were performed using SPSS (version 24.0; SPSS, Inc., Chicago, IL, USA). The normality of the data was assessed using the Shapiro–Wilk test. Continuous variables are presented as mean ± standard deviation (SD). Paired measurements from the two devices were compared using the paired t-test for variables with normal distribution and the Wilcoxon signed-rank test for those without normal distribution. The agreement between the devices was evaluated using Bland–Altman analysis, which involved calculating the mean difference (bias) and 95% limits of agreement (LoA). Device reliability was assessed using the Intraclass Correlation Coefficient (ICC), employing a two-way random-effects model with absolute agreement. The association between the measurements from the two devices was further examined using the Pearson correlation coefficient. Statistical significance was set at *p* < 0.05.

## Results

The study included the right eye of 60 patients scheduled to undergo cataract surgery, comprising 28 (46.7%) women and 32 (53.3%) men. The mean age of the participants was 61.98 ± 10.69 years, with an age range of 40–82 years. The mean preoperative AL was 24.22 ± 1.91 mm for REVO FC and 24.23 ± 1.91 mm for AL Scan, with no statistically significant difference observed (Table 1, *p* = 0.507). The mean preoperative ACD was 3.25 ± 0.40 mm for the REVO FC and 3.23 ± 0.41 mm for the AL Scan, with no statistically significant difference between the two devices in terms of ACD measurements (Table 1, *p* = 0.218). However, statistically significant differences were observed in keratometric measurements. The flat keratometry (K1) values were 44.31 ± 1.65 D for the REVO FC and 43.54 ± 1.68 D for the AL Scan (2.4 mm), with a mean difference of 0.76 D (*p* < 0.001). Similarly, the steep keratometry (K2) values were 45.51 ± 1.94 D and 44.57 ± 1.79 D, respectively, with a mean difference of 0.94 D (*p* < 0.001). The REVO FC produced steeper keratometric values than the AL Scan. In relation to the J0 and J45 vector values, the J0 value was recorded as -0.17 ± 0.61 for the REVO FC and − 0.16 ± 0.58 for the AL Scan, with a mean difference of -0.008. No statistically significant differences were observed between the groups (*p* = 0.889). The J45 values were − 0.09 ± 0.50 and 0.02 ± 0.40, respectively, with a significant mean difference of − 0.111 (*p* = 0.028). All p-values are summarized in Table 1. A strong positive correlation was observed between the two devices for all the evaluated parameters. The axial AL demonstrated an almost perfect correlation (*r* = 0.998; *p* < 0.001). Similarly, ACD showed a strong correlation (*r* = 0.945, *p* < 0.001). Keratometric measurements also revealed strong correlations with *r* = 0.980 (*p* < 0.001) for K1, and *r* = 0.924 (*p* < 0.001) for K2. Pearson correlations and intraclass correlation coefficients (ICC) between Revo FC and AL Scan are summarized in Table 2.

**Table 1:** Biometry values for Revo FC and AL Scan

Parameter	Revo FC Mean ± SD	Range	AL Scan Mean ± SD	Range	Mean difference	*P* value
AL (mm)	24.22 ± 1.91	21.99–30.40	24.23 ± 1.91	21.98–30.45	-0.01	0.507ᵃ
ACD (mm)	3.25 ± 0.40	2.54–4.31	3.23 ± 0.41	2.50– 4.12	0.02	0.218ᵃ
K1 (D)	44.31 ± 1.65	40.75–48.29	43.54 ± 1.68	40.13–47.43	0.76	< 0.001ᵃ
K2 (D)	45.51 ± 1.94	41.40–49.30	44.57 ± 1.79	40.66–48.13	0.94	< 0.001ᵃ
J0	-0.17 ± 0.61	-2.11/ 1.47	-0.16 ± 0.58	-2.37/1.48	-0.008	0.889ᵃ
J45	-0.09 ± 0.50	-1.98 ± 0.67	0.02 ± 0.40	-1.33 ± 1.09	-0.111	0.028ᵃ

SD: standard deviation, AL: axial length, ACD: anterior chamber depth, K1: flat keratometry, K2: steep keratometry ᵃ Paired t-test

**Table 2:** Pearson Correlations and Intraclass correlation coefficients (ICC) between Revo FC and AL Scan

Parameter	Pearson *r*	ICC
AL	0.998	0.998
ACD	0.945	0.945
K1	0.980	0.882
K2	0.924	0.808
J0	0.707	0.752
J45	0.667	0.665

AL: axial length, ACD: anterior chamber depth, K1: flat keratometry, K2: steep keratometry

Bland–Altman analysis demonstrated excellent agreement for AL measurements, with a mean difference (bias) of − 0.01 mm and narrow 95% LoA (− 0.245 to 0.225 mm). For ACD, the mean difference was 0.02 mm, with a slightly wider 95% LoA (− 0.244 to 0.288 mm), indicating acceptable agreement.

In contrast, the keratometric parameters revealed clinically significant discrepancies. K1 showed a mean difference of 0.76 D, with a 95% LoA ranging from 0.11 to 1.42 D, indicating a systematic bias between the devices. K2 demonstrated an even larger mean difference of 0.94 D and a substantially wider 95% LoA (− 0.51 to 2.39 D), suggesting a poor agreement and limited interchangeability.

**Fig. 1:**
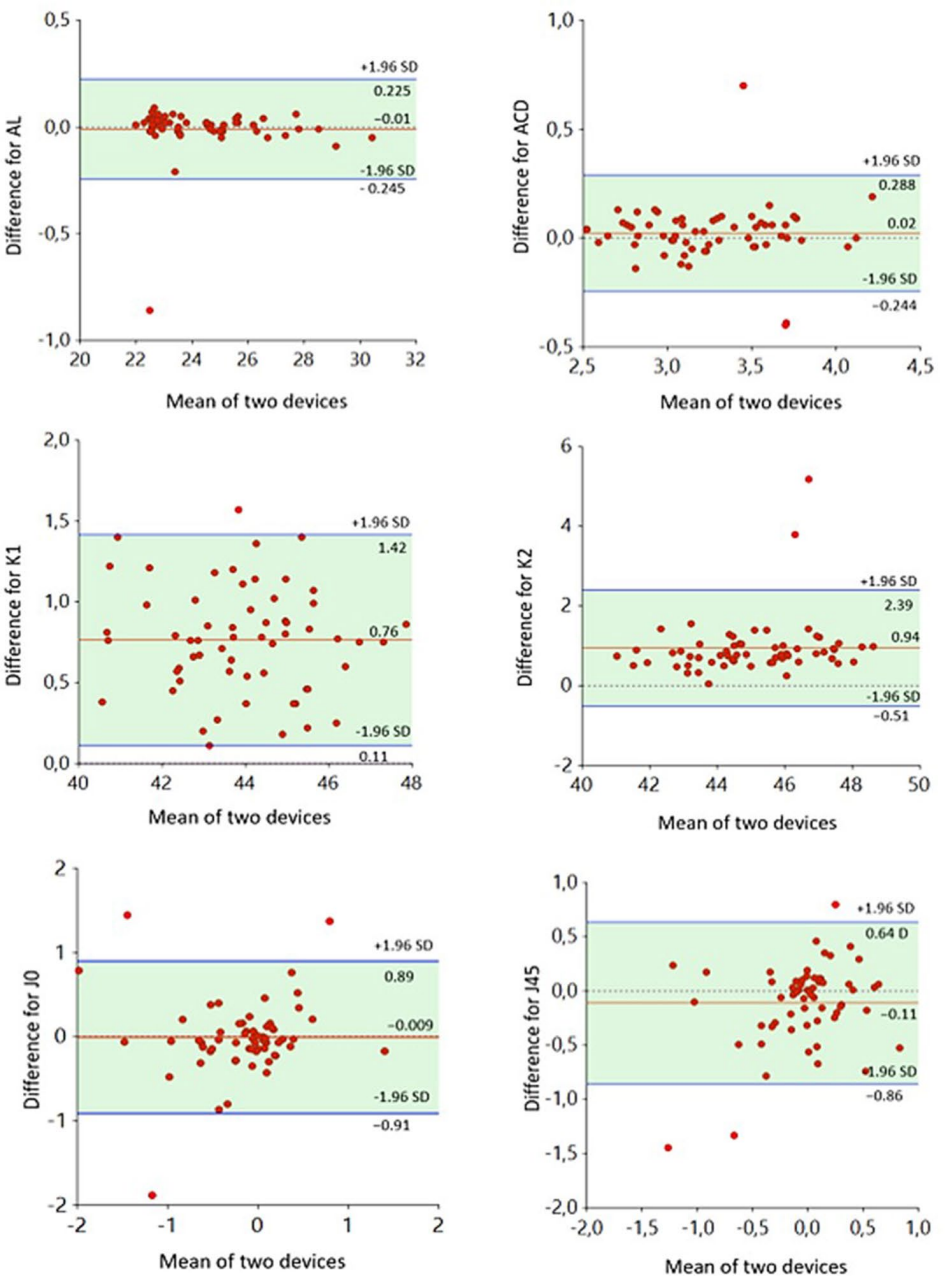
Bland-Altman plots showing agreement between REVO FC and AL Scan at AL, ACD, K1, K2 keratometric measurements, J0 and J45 vectors. The middle red line presents the mean difference, the bottom and the top blue lines show the lower and upper 95% limits of agreement

To overcome the limitations of the linear axis analysis, astigmatic data were transformed into vector components (J0 and J45). For J0, Bland–Altman analysis demonstrated minimal bias (− 0.009), with limits of agreement ranging from − 0.910 to 0.890. A moderate positive correlation was observed (*r* = 0.707, *p* < 0.001), with an intraclass correlation coefficient indicating moderate agreement. For J45, the mean difference was − 0.110, with limits of agreement between − 0.860 and 0.640. Correlation analysis revealed a moderate positive correlation (*r* = 0.667, *p* < 0.001), and agreement was also moderate. These findings indicate that, although the systematic bias is minimal, the variability remains considerable for both vector components. Despite minimal systematic bias, the relatively wide limits of agreement in the vector components suggest that the two devices should not be used interchangeably for astigmatism assessment in precision-demanding applications, such as toric IOL calculation. Bland-Altman plots showing the agreement between the REVO FC and AL Scan measurements at the AL, ACD, K1, K2, J0, and J45 vectors are presented in Fig. 1.

## Discussion

The present study determined that the AL Scan and REVO FC SD-OCT devices exhibited excellent concordance in AL measurements, with a mean difference of − 0.01 mm and a narrow 95% LoA ranging from − 0.245 to 0.225 mm. In terms of ACD, the mean difference was 0.02 mm, with a slightly wider 95% LoA of − 0.244 to 0.288 mm, indicating a clinically acceptable but less robust agreement than AL measurements. However, the keratometric parameters revealed clinically significant discrepancies. The REVO FC measurements indicated steeper keratometric values. Specifically, K1 exhibited a mean difference of 0.76 D, with a 95% LoA ranging from 0.11 to 1.42 D, suggesting a systematic bias between the devices. K2 demonstrated an even larger mean difference of 0.94 D and a substantially wider 95% LoA of − 0.51 to 2.39 D, indicating poor agreement and limited interchangeability between the two instruments. The evaluation of the J0 and J45 vector components, both vector components revealed moderate correlation and J45 revealed significant differences, suggesting that the two devices should not be used interchangeably for keratometric and astigmatism measurements.

With the advent of the IOL Master (Carl Zeiss Meditec, Jena, Germany) in the late 1990s, the field of non-contact (optical) biometry for intraocular lens calculation in cataract surgery was initiated. This development was followed by the introduction of the Lenstar (Haag-Streit AG, Koeniz, Switzerland) in 2008 and AL Scan (Nidek Co., Ltd., Gamagori, Japan) in 2012. Subsequently, in 2015, the introduction of swept-source OCT with the IOL Master 700 (Carl Zeiss Meditec, Jena, Germany) enabled biometric measurements using OCT devices [12–15]. The literature extensively discusses the repeatability and interchangeability of biometry devices in achieving more precise refractive outcomes in cataract surgery, as well as in premium and toric IOL applications.

Srivannaboon et al. assessed the concordance between the IOL Master 500 and AL Scan in 137 eyes from 81 patients. Both devices demonstrated high repeatability and reproducibility across all ocular biometry measurements (ICC, 0.87-1.00). With the exception of the WTW diameter (ICC, 0.44), the agreement between the biometers was also substantial (ICC, 0.98–0.99). The intraocular lens powers calculated using the Holladay 1 formula were comparable between the two biometers, with a mean difference of only 0.26 D, which is less than the increment in the IOL power step (0.50 D) (ICC, 0.994) [16]. The AL Scan device quantifies keratometric values by projecting double-mire rings onto the corneal surface at the 2.4 mm and 3.3 mm zones. The IOLMaster 500 determines keratometry values by projecting six spots of light onto the cornea within the central 2.5 mm zone. Srivannaboon et al. also determined that the keratometric data from the IOL Master at 2.5 mm exhibited closer agreement (LoA − 0.26 to 0.44; ICC 0.996) with the K values measured by the AL scan at 2.5 mm. In contrast, a slightly broader range of agreement (LoA − 0.32 to 0.48; ICC 0.995) was noted compared to the AL Scan measurements at 3.3 mm.

Given that the IOL Master utilizes the 2.5 mm zone and many IOL manufacturers employ keratometric data measured by the AL Scan at the 2.4 mm zone for IOL calculations, we utilized the data from this zone in our study. In the present study, we hypothesize that the lower agreement observed in keratometric measurements with the REVO FC SD-OCT device may be partly related to differences in the keratometric measurement zone, as this device performs keratometry using OCT-derived corneal data from the 3.0 mm zone. Previous studies have shown that variations in keratometric measurement diameters and acquisition principles may influence agreement between biometers and contribute to systematic differences in corneal power measurements [17, 19]. Therefore, the discrepancy observed between the REVO FC and AL Scan devices may reflect differences in both measurement zone and underlying measurement technology rather than true differences in corneal curvature.

In another comparative study of the AL Scan and IOL Master, Huang et al. reported a high level of agreement between the two devices concerning AL measurements, with 95% LoA ranging from − 0.12 to 0.10, and ACD measurements, with 95% LoA from − 0.28 to 0.35. The study also compared keratometric data from the IOL Master with that obtained by the AL Scan at diameters of 2.4 mm and 3.3 mm. A significant finding was that the keratometric values obtained by the AL Scan using a 2.4 mm diameter exhibited better concordance with the IOL Master measurements than those obtained using a 3.3 mm diameter. Specifically, the 95% LoA for Km (2.4 mm) was − 0.44 to 0.54 D, whereas for Km (3.3 mm), it was − 0.56 to 0.61 D [17]. The findings indicate that variations in the width of the keratometric zone and the measurement methodologies employed by biometry devices may lead to inconsistent results between biometry devices.

Huang et al. also conducted a comparative analysis of the AL Scan and an anterior segment SD-OCT device (Visante AS-OCT, Carl Zeiss Meditec, Dublin, USA) with respect to corneal thickness and ACD [18]. Their findings indicated that the ACD measurements obtained from the AL Scan and Visante SD-OCT were comparable, with a mean difference of -0.02 mm (𝑃 > 0.05), and demonstrated good agreement, as evidenced by the 95% LoA ranging from − 0.15 to 0.12 mm. In the present study, we observed a mean difference of 0.02 mm between the AL Scan and REVO FC SD-OCT device ACD measurements, albeit with a broader 95% LoA range from − 0.24 to 0.28 mm. Our findings concerning ACD align with those of Huang et al., who demonstrated that the AL Scan and SD-OCT devices are interchangeable with respect to ACD.

The AL Scan was compared with the IOL master 700 (Carl Zeiss Meditec, Jena, Germany), which is a swept-source OCT (SS-OCT) device, in a study by Chan in 52 eyes of 52 patients who were candidates for cataract surgery [19]. Both devices provided excellent repeatability for AL, ACD, and keratometry measurements in patients with cataracts. The AL measurements showed no statistically significant difference between the devices, with a mean difference of 0.014 mm and narrow limits of agreement, indicating strong concordance. In contrast, keratometry values obtained with the AL Scan were consistently steeper, with mean differences of approximately − 0.196 D (2.4-mm zone) and − 0.144 D (3.3-mm zone), reflecting a systematic bias between the devices. Similarly, ACD measurements exhibited a small but statistically significant difference, with the AL Scan measuring slightly deeper values (mean difference, 0.017 mm). Despite these statistically significant differences in keratometry and ACD, the magnitude of the discrepancies was considered clinically negligible. Overall, although AL measurements can be regarded as interchangeable between devices, caution may be required when interpreting keratometry and ACD.

A recent study comparing AL measurements obtained using the PCI biometer Myopia Master (Oculus Optikgerate; Wetzlar, Germany) and SD-OCT REVO FC 80 (Optopol Technology; Zawiercie, Poland) demonstrated excellent agreement between the two technologies [11]. The mean difference between the devices was minimal (0.002 mm) and not statistically significant, with a very high concordance correlation coefficient (CCC = 0.9995), indicating a near-perfect correlation. Bland–Altman analysis revealed narrow limits of agreement ranging from − 0.061 to 0.065 mm, supporting a strong consistency between the measurements. Additionally, both devices showed equally high repeatability, with an ICC of 0.999. These findings suggest that PCI- and SD-OCT–based biometers provide comparable and highly reliable AL measurements. In the present study, we also found a perfect correlation and agreement between the PCI biometer AL Scan and REVO FC SD-OCT in terms of AL measurements.

The current study is subject to certain limitations, notably a small sample size and the restriction of participants to individuals over 40 years of age with cataracts. Future research should include larger studies involving cohorts from a more diverse range of age groups.

## Conclusion

The PCI-based biometry AL Scan and the SD-OCT device REVO FC demonstrate excellent agreement and interchangeability concerning AL and ACD measurements. However, keratometric measurements reveal significant bias and a wide limit of agreement. Consequently, these devices should not be used interchangeably for keratometric values.

## Data Availability

No datasets were generated or analysed during the current study.
